# Preface to the theme issue ‘Science into the next millennium: 25 years on’

**DOI:** 10.1098/rsta.2023.0385

**Published:** 2025-05-08

**Authors:** John Dainton

**Affiliations:** ^1^ Cockcroft Institute, Warrington, England, UK

In January 2000, *Philosophical Transactions A* published three theme issues, entitled ‘*Science into the next millennium: young scientists give their individual visions of the future*’ [1]. One issue covered astronomy and earth sciences,^1^ the second covered mathematics, physics and engineering^2^ and the third covered chemistry and biological physics.^3^ The issues were compiled by Professor J. Michael Thompson, FRS, who had recently taken over as editor-in-chief of the journal. A quarter of a century later, the journal invited some of the original authors to contribute a follow-up to their submissions of 25 years ago, and I was delighted to oversee this as my final job during my tenure as editor-in-chief.

These contributions reveal not only a record of achievement with the usual perspectives of added insight and significance, but they also illustrate the degrees to which, with the passage of time, progress in some of these examples of ‘cutting-edge’ scientific research is reported as having been made, or is not. The contributions are concerned with specific outcomes, with overviews of how matters have progressed and with how challenges have steered work in new directions following new aspirations. As is to be expected, serendipity and predictability sit side by side, and some invitations to contribute to this ‘follow up’ theme issue were declined, presumably because developments after 2000 were unfruitful. Such an outcome is always expected in the very best scientific research into the unknown, for otherwise, it wouldn’t of course be the very best research!

## 1. Adieu

It has been a privilege to be the editor-in-chief of *Philosophical Transactions A* for 7 years, following some time also serving on the editorial board. Apart from its historical pedigree, the like and match of which I am not aware is to be found anywhere else on the planet, this privilege has extended also to the delightful challenges of opportunities to inform oneself of important and significant progress in a broad range of scientific disciplines, away from the day-to-day obsession with one’s own work. Moreover, this self-improvement matters when the few instances in which the peer review produces uncertainty, even constructive controversy, and the need for arbitration ends up on the editor-in-chief’s screen.

Throughout my tenure, I have been fortunate to have worked with two excellent commissioning editors: first Bailey Fallon and then Alice Power. Alice’s time with the journal included the challenging period of the COVID-19 pandemic, the consequences of which posed throughout unusually demanding commitment to maintaining the functionality of the journal and dealing with many unwanted, unhelpful and time-consuming consequences. I would also like to put on record my appreciation and gratitude to Helen Eaton, Senior Commissioning Editor. The guidance and support that her wisdom and experience always bring to *Philosophical Transactions A*, alongside her responsibilities for other journals, are much appreciated. Her input is always unobtrusive and significant because it is carefully considered, given the prevailing circumstances of the matter in hand. I have benefitted many times from her remarks and advice, as I am sure others have, and I thank her for them.

It is also highly appropriate to record and acknowledge the commitment of the Editorial Board of *Philosophical Transactions A*. Its membership is the backbone to ensuring that the journal is publishing good content, as well as providing invaluable advice on many other concerns that arise as time passes. The board ensures that the interests of the disparate scientific communities and their research cultures that are essential for a journal with the scientific scope of *Philosophical Transactions A* are always at the forefront of decision-making wherever necessary. In this respect, I am grateful for unforgettable stimulating memories of one-to-one discussions with many who, while serving on the board, also have managed to find time to attend in-person annual meetings at Carlton House Terrace. I am convinced that together they constitute a unique source of expertise, which is vital for very many aspects that make up the continuing excellence of *Philosophical Transactions A*, and I hereby thank them for their input over the years. Here also one must recognize the substantial benefits ensuing from the emergence of video calling technologies during the COVID-19 pandemic and how their continuing availability has rejuvenated annual board meetings and has enabled remote attendance of members at more specialized ad hoc meetings when they have been necessary.

When I joined the journal, other than the usual demands of writing and refereeing putative publications in my own research field, I had little or no experience of the workings of a scientific journal. Thanks to my years as an editor-in-chief, I can now claim better, but only because of what I have now seen, heard and learned observing the staff of the Royal Society’s publishing arm. The challenges that they have faced and have reported at the Society’s publication committee meetings have demonstrated to my untutored eyes and ears an immensely professional outfit doing an immensely important, outward-facing activity for the benefit of science everywhere. My experiences have left in my consciousness an enhanced sense of commitment to ‘not for profit’ publishing enterprises, which confirms my long-held belief that scientific publishing deserves at least that, and Royal Society publishing exemplifies it at its very best.

I must also mention the essential contributions of the guest editors. They play a special and crucial role in sustaining the modus operandi of PTRSA from the beginning, the proposal for a theme issue with or without a discussion meeting, to the end, and its publication. I am pleased that their experiences with the journal have usually been positive and, in many cases, have benefitted their own careers. Of course, the guest editors’ successes depend on the quality of those who submit contributions, so credit and gratitude are also due there!

So now all that remains is for this physicist to wish my successor, the distinguished chemist Professor Sir Richard Catlow FRS, well as he takes up the *Philosophical Transactions A* ‘baton’. If he has as much enjoyment, even fun, as I have had in the job, I am sure that he will take the journal to new heights.

## Data Availability

This article has no additional data.

## Footnotes

^1^

https://royalsocietypublishing.org/toc/rsta1996/1999/357/1763

^2^

https://royalsocietypublishing.org/toc/rsta1996/2000/358/1765

^3^

https://royalsocietypublishing.org/toc/rsta1996/2000/358/1766

## References

[1] Thompson JMT. 1999 Philosophical Transactions into the 21st Century: an editorial. Phil. Trans. R. Soc. Lond. A (ed. JMT Thompson), **357**, 3187–3195. (10.1098/rsta.1999.0488)

